# Case Report: Recovery of consciousness ahead of MRI image lesion information in cerebral fat embolism syndrome

**DOI:** 10.3389/fmed.2025.1566578

**Published:** 2025-06-05

**Authors:** Zhixiong Zhuang, Yan Bo, Yunpin Pan, Jianfeng Huang

**Affiliations:** ^1^Intensive Care Unit, Ningbo Sixth Hospital, Ningbo, China; ^2^Department of Medicine, Northwest Minzu University, Lanzhou, China

**Keywords:** cerebral fat embolism syndrome, consciousness disorder, recovery cycle, MRI lesions, case report

## Abstract

**Background:**

The absence of definitive international guidelines for the diagnosis and treatment of fat embolism syndrome (FES) has prompted clinicians to undertake independent research. The admission of a patient with suspected cerebral FES (CFES) prompted clinicians to engage in self-directed learning.

**Case summary:**

We presented a case of a 28-year-old male patient who had developed CFES as a complication of a fracture. The initial magnetic resonance imaging (MRI) scan revealed the presence of scattered, multiple punctate lesions in the majority of the cerebral white matter. Following a series of treatments, including supportive care, the final MRI scan (on the fifth day) demonstrated that the majority of lesions had either been resorbed or were undergoing resorption, with a small number of lesions demonstrating fusion and an increase in size. However, the patient’s impaired consciousness was successfully restored 5 days after the onset of the disease.

**Conclusion:**

MRI findings should serve as the foundation for diagnosing CFES, rather than being used as a criterion for evaluating discharge. We proposed that MRI findings of “star pattern” and “vasogenic edema” represented the optimal imaging criteria for diagnosing CFES. The timing of the diagnosis of CFES can be utilized as a validation measure for the diagnosis of CFES, which was conducive to the early and complete recovery from consciousness disorders. Moreover, we found the lesion information from MRI images lags behind the rate of recovery in the level of consciousness. The clinician can consider that the cerebral fat embolism syndrome has reached the therapeutic expectation when the patient’s level of consciousness is restored. The patient can then be asked to be followed up after discharge from the hospital, and the end point of the follow-up period can be indicated by observing the complete disappearance of the lesion information shown on MRI.

## 1 Introduction

Cerebral fat embolism syndrome (CFES) represents a rare but significant complication of fractures (1). Furthermore, misdiagnosis is a significant concern, as the condition is associated with considerable disability (2). Previous methods of determining improvement in CFES relied on the degree of MRI lesion resorption and Glasgow Coma Scale (GCS) (3). However, the available literature does not provide further details regarding the time required for the improvement of impaired consciousness and the degree of MRI lesion resorption in patients with CFES. In this case, the brain MRI of a patient with CFES demonstrated resorption of the majority of scattered, multiple lesions. However, a small number of lesions exhibited signs of fusion and increased size. Interestingly, the patient exhibited complete recovery from impaired consciousness. This finding highlights the importance of continued observation and monitoring in cases of CFES.

## 2 Methods

The study used a case report research design.

The present case reported details a case of cerebral fat embolism syndrome after a car accident. The present clinical hypothesis posited that the time to recovery following treatment in patients with cerebral fat embolism syndrome should be prioritised over the time to resorption of embolic lesions.

This case report adheres to the CARE guidelines for human participant case studies and was conducted with full ethical compliance, which was developed by Riley et al. (4).

## 3 Results

### 3.1 Patient information

A 28-year-old male patient presented at the emergency department following a traffic accident that had occurred 5 h prior. He was noted to have bilateral lower extremity pain and limited mobility upon admission. On that day, the patient was observed to be coherent and reported no past history of traumatic surgery or other significant medical conditions.

Upon arrival at the emergency department, the patient was scheduled for imaging. The results of the radiographic examination indicated the presence of a fracture of the left mid-femur, a fracture of the left inferior patella, a fracture of the right ankle, and a fracture of the right fibula. A comprehensive computed tomography scan of the head and chest did not yield any noteworthy abnormalities. The patient was subsequently referred to the orthopedic department. At that time, the first physician provided a proposed treatment plan, which included “traction braking of the left lower extremity.” On the following day, the patient abruptly manifested signs of disorientations. The physician neglected to take into account the patient’s diminished level of consciousness. Cardiac monitoring demonstrated a finger pulse oximetry (SPO₂) value of 88%. After administration of oxygen, the patient’s SPO₂ returned to normal, yet there was no restoration of the level of consciousness. A brain CT and a brain MRI were scheduled at that time; however, CT did not disclose any abnormal manifestations and was terminated due to the patient’s sudden foaming at the mouth during MRI. At that time, the patient was administered symptomatic treatment, which resulted in an improvement in the sudden symptoms. By the fourth day, the patient’s level of consciousness had further deteriorated and he was now comatose. The GCS at that point was 7, reflecting the presence of both aphasia and stabbing eye movements, in addition to stabbing avoidance.

**Figure 1:**
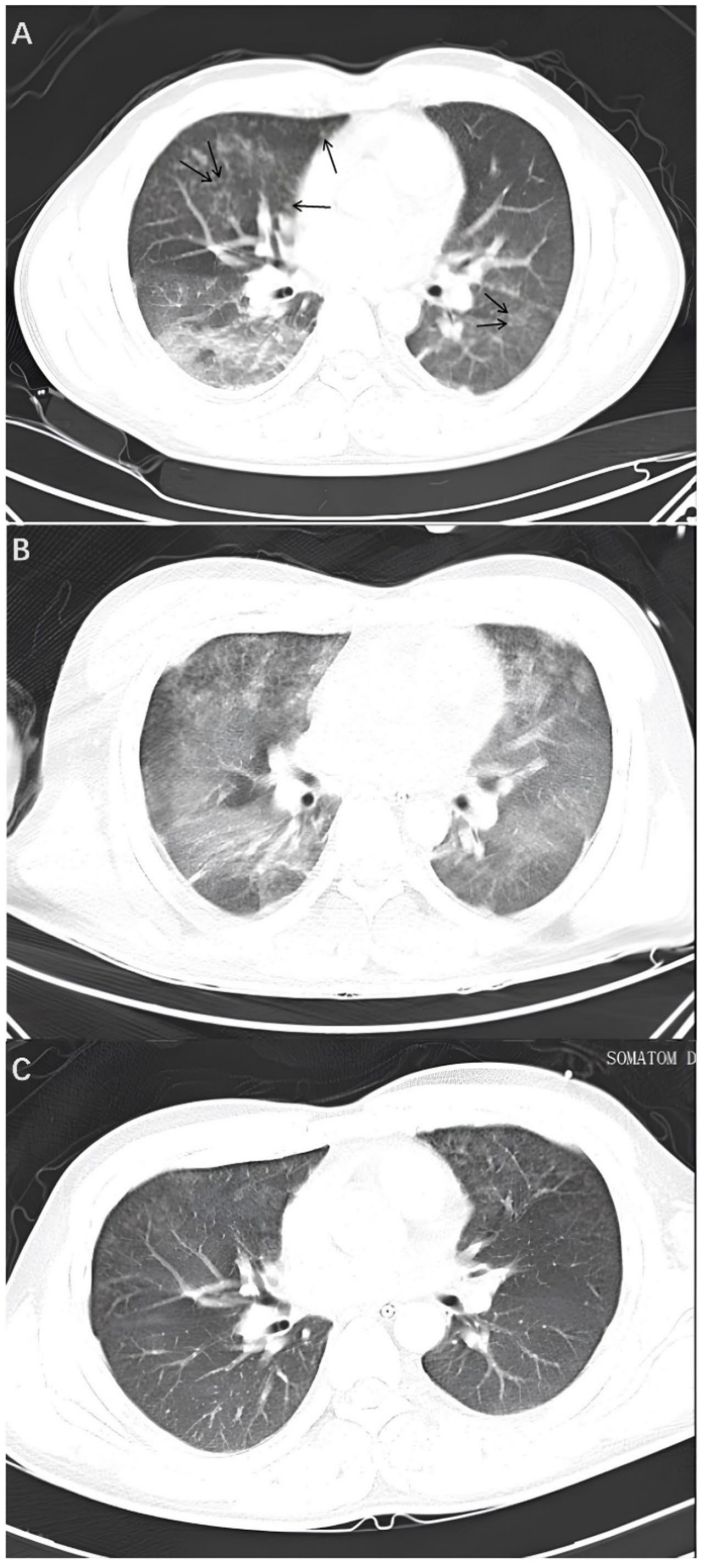
Lung CT. **(A)** On the fourth day, a lung CT scan revealed the presence of multiple ground-glass opacity exudative lesions in both lungs, in addition to several minute nodules exhibiting an irregular distribution (illustrated by the black arrows). **(B)** On the sixth day, the lung CT scan indicated that the lung lesions had worsened, with extensive and diffuse ground-glass exudative lesions in both lungs. **(C)** On the tenth day, the lung CT scan showed that the ground-glass opacities in both lungs had significantly decreased.

A physical examination revealed that the patient was breathing at a rate of 23 times per minute, which is above the norm. A CT scan of the lungs revealed the presence of multiple ground-glass shadow exudative lesions in both lungs, as well as a multitude of minute nodules distributed in an irregular pattern (Figure 1A). The patient was transferred to the intensive care unit (ICU) due to the severity of his illness.

### 3.2 Clinical findings

**Figure 2:**
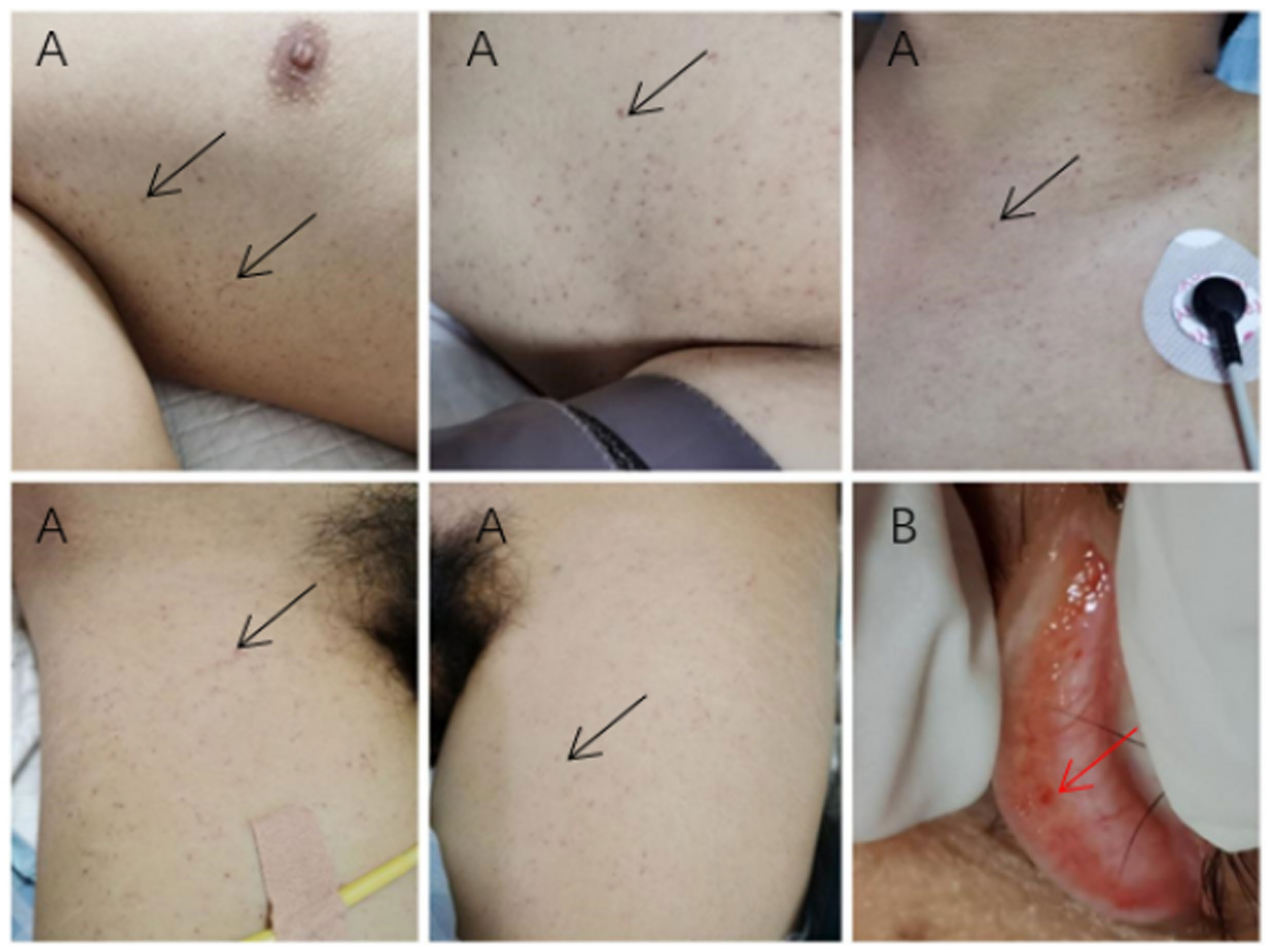
Cutaneous and conjunctival manifestations. **(A)** Subcutaneous petechiae in the bilateral axillae and anterior chest (black arrows). **(B)** Conjunctival hemorrhagic spots (red arrow).

Upon examination by the ICU physician, multiple red or brown hemorrhagic spots were observed on the skin and conjunctiva (Figure 2). Conjunctival hemorrhages were localized to the bulbar conjunctiva (Figure 2B), distinct from periorbital trauma-related ecchymosis. The hemorrhagic spots on the skin, pulmonary symptoms, pulmonary CT manifestations, and impaired consciousness, which were not related to trauma, met the diagnostic criteria set forth by Gurd (5, 6).

### 3.3 Diagnostic assessment

Upon examination by the ICU physician, multiple red or brown hemorrhagic spots were observed on the skin and conjunctiva (Figure 2). The hemorrhagic spots on the skin, pulmonary symptoms, pulmonary CT manifestations, and impaired consciousness, which were not related to trauma, met the diagnostic criteria set forth by Gurd (5, 6).

### 3.4 Therapeutic intervention

The patient was administered a comprehensive treatment regimen, which included measures to maintain airway patency, oxygen supplementation, and the administration of an 80 mg intravenous methylprednisolone injection, gradually tapered until discontinued on the eighth day. Additionally, the patient received a 200,000 U intravenous injection of ulinastatin, a 50 g intravenous human albumin injection, and a 4,100 IU intravenous low molecular heparin calcium injection, all administered over a three-day period.

### 3.5 Follow-up and outcomes

**Figure 3:**
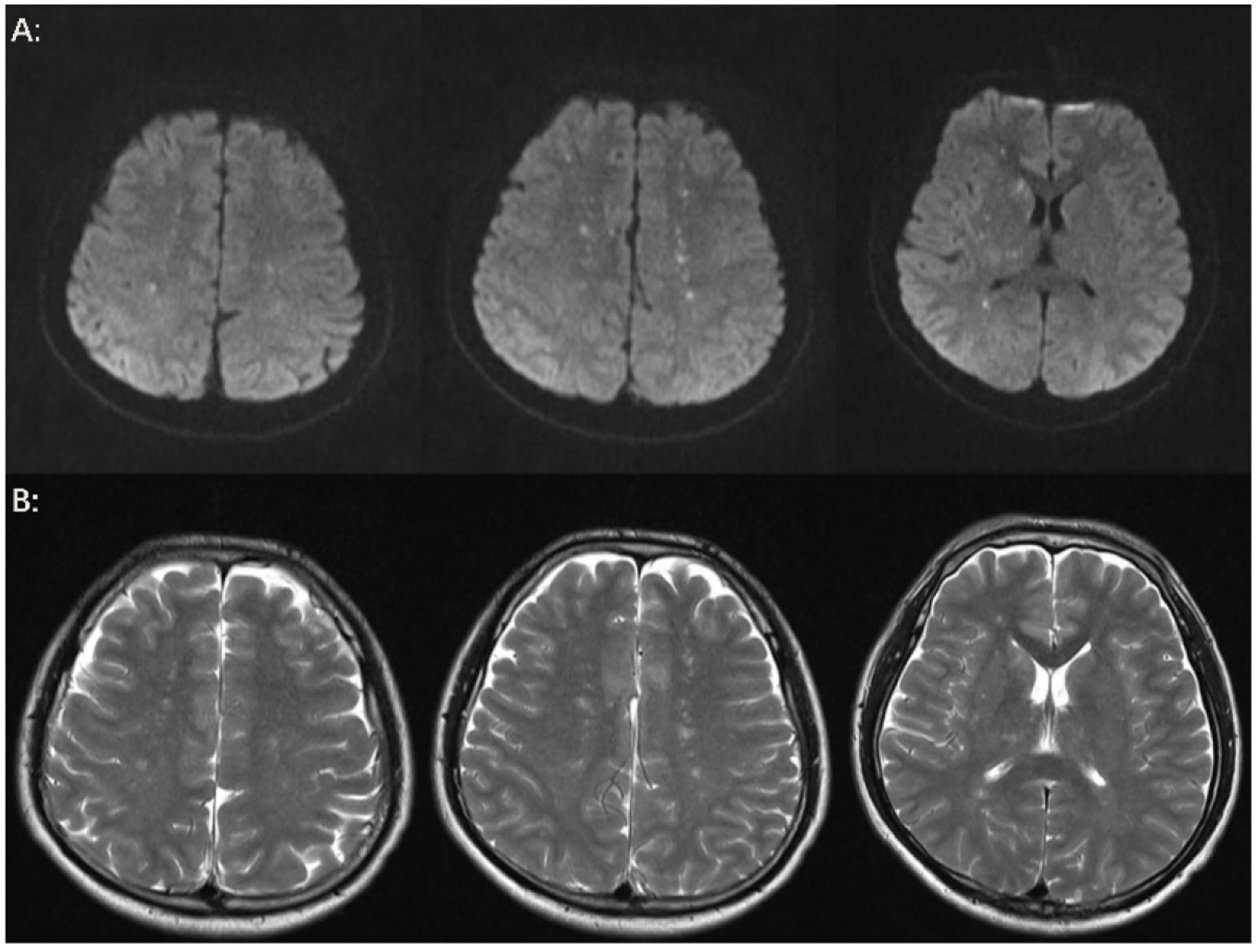
Brain MRI. **(A)** On the fifth day, the results of the brain MRI-DWI indicated the presence of multiple punctate lesions exhibiting high or slightly elevated signal intensity in various regions, including the bilateral centrum semiovale, the basal ganglia, the bilateral thalamus, the bilateral frontal lobes, and the bilateral parietal lobes. The majority of lesions were distributed in the cerebral white matter, while a few were located in the cerebral gray matter, exhibiting a starry sky sign. Multiple punctate high or slightly high signals on DWI indicate restricted diffusion, which may be accompanied by micro-fat embolism and cytotoxic edema. **(B)** On the fifth day, brain MRI-T2WI demonstrated multiple punctate high or slightly high signal lesions in the bilateral centrum semiovale and basal ganglia region and bilateral thalamus, suggesting the possibility of vasogenic edema.

**Figure 4:**
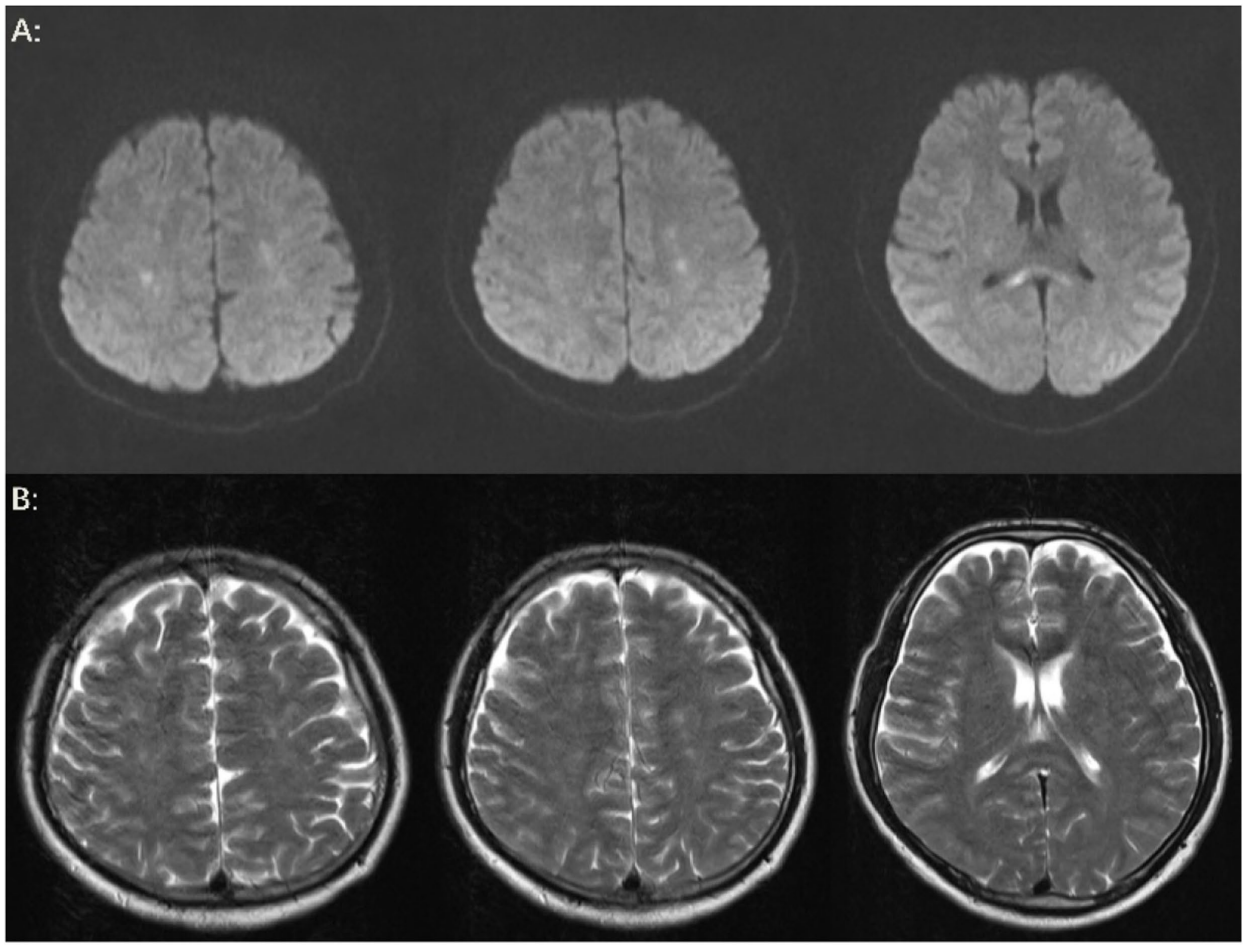
Brain MRI. **(A)** On the twelfth day, the results of the brain MRI-DWI indicated that the majority of lesions had been absorbed following treatment, with a few lesions showing evidence of fusion and enlargement. Additionally, a new cord-like high signal was observed in the splenium of the corpus callosum. **(B)** On the twelfth day, the brain MRI-T2WI findings also suggested that the majority of lesions had been absorbed after treatment, and a new cord-like high signal was noted in the splenium of the corpus callosum.

The patient’s level of consciousness remained stable following the administration of the aforementioned treatment regimen. On the fifth day, a brain MRI with diffusion-weighted imaging (DWI) (Figure 3A) demonstrated scattered and multiple speckled high/slightly high signal foci in the brain, with limited diffusion. This finding resembled the star sign, indicating that the fat emboli primarily affected the cerebral white matter and caused cytotoxic cerebral edema. It is therefore plausible that the fat embolus may have caused cytotoxic cerebral edema. An MRI in T2-weighted imaging (T2WI) (Figure 3B) also demonstrated scattered and multiple speckled high/slightly high signals within the brain, which differed from those observed in DWI. The increased signals in T2WI indicated the potential for vasogenic cerebral edema. A review of the lung CT on the sixth day indicated a further increase in lesions in both lungs (Figure 1B). However, the patient’s clinical manifestations showed improvement, and the treatment plan remained unchanged. On the seventh day, the patient exhibited a complete restoration of his level of consciousness. A follow-up lung CT on the tenth day demonstrated notable absorption of exudative lesions in both lungs (Figure 1C). A brain MRI on the twelfth day indicated that the majority of lesions had been absorbed, with a few exhibiting increased size after fusion (Figures 4A,B). Quantitative analysis of MRI lesions revealed a reduction in total lesion count from 42 (Figure 3A) to 18 (Figure 4A), with mean lesion diameter decreasing from 3.2 mm to 1.8 mm. However, residual lesions in T2WI (Figure 4B) persisted, suggesting ongoing vasogenic edema. Long-term follow-up MRI is recommended to monitor lesion evolution. Following 12 days of continuous observation and treatment, the patient was discharged from the hospital.

### 3.6 Timeline

**Figure 5:**
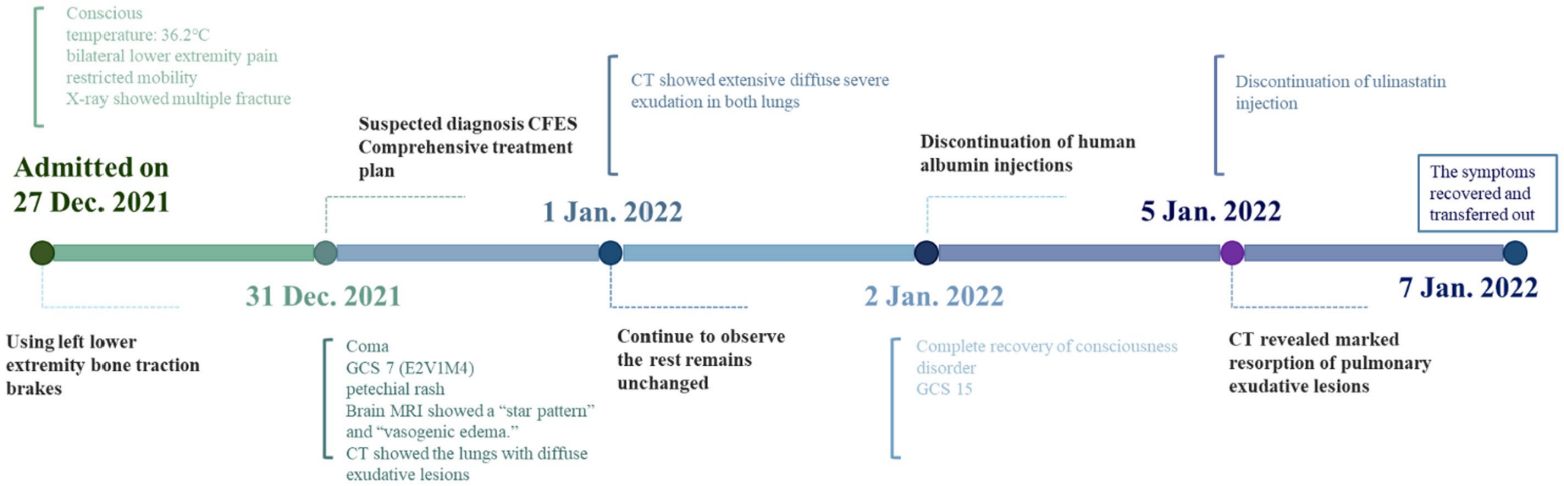
Timeline. This is a treatment timeline drawn by Figdraw with milestone time points for CFES treatment.

The timeline of the patient’s clinical course described above is presented in Figure 5.

## 4 Discussion

### 4.1 Strengths and limitations

It is proposed that the “star pattern” and “angioedema” as interpreted from MRI images should be used as the basis for diagnosing CFES. Conversely, this case contributes novel insights into the prognosis of CFES patients. Specifically, the lesion information derived from MRI images does not align with the level of consciousness observed in actual CFES patients. Moreover, the lesion information from MRI images lags behind the rate of recovery in the level of consciousness. Consequently, it is unwarranted for clinicians to utilize MRI image results as a criterion for discharging CFES patients based on their level of consciousness.

The limitation of this case report is the lack of internationally recognized and reliable diagnostic criteria for CFES. Additionally, Gurd previously suggested the detection of fat globules in urine and fat globules in sputum as potential indicators of CFES. While this may appear reasonable, it is not currently applicable in clinical practice. It is hoped that future clinical guidelines for CFES will be universally applicable.

In addition, a case of cerebral fat embolism syndrome based on a review of clinical practice may suggest that there is no correlation or parallelism between the time of recovery of the patient’s level of consciousness and the time of MRI lesion uptake. This may have been a fortuitous circumstance (4), such as the young age of this patient. The age factor may be responsible for the chance phenomenon. It is important to note that the rapid recovery observed in this case may be attributed to the patient’s young age and absence of comorbidities. Older patients with reduced neuroplasticity or pre-existing cerebrovascular conditions may exhibit slower or incomplete recovery. Future studies should include a broader age range to validate the generalizability of these findings. If there is a need to investigate the relationship between the time to recovery of the patient’s level of consciousness and the time to MRI lesion uptake, a prospective cohort study with standardised research protocols should be used.

### 4.2 Literature review

Bo et al. (6, 7) believes that the necessary literature review can enhance the reliability of new findings in the case. Necessary literature reviews are widely used not only in clinical studies (8) but also in systematic reviews (9–11). This is where we run the necessity of literature review. The occurrence of fat embolism syndrome has been documented in the scientific literature, with fracture trauma identified as the primary etiology (12, 13). Upon initial presentation to the hospital, patients with such fractures are typically treated symptomatically, with a focus on fracture repair surgery. While this approach may meet the patient’s expectations for the visit, the accumulation of fat particles at the fracture site can gradually increase, entering damaged small vessels and leading to the development of symptoms predominantly affecting the nervous system or respiratory system. A scoping review indicated that the clinical manifestations of FES can progress rapidly, with patients transitioning from shortness of breath and hypoxemia to respiratory failure within a single day. The use of mechanical ventilation in such patients has been demonstrated to prevent 44% of adverse outcomes (14). The challenge of diagnosing cerebral FES is further compounded by evidence indicating that the initial manifestation of FES in over 50% of patients is confusion and status epilepticus (15).

A case report revealed the absence of a bruising rash at the onset of cerebral fat embolism syndrome (FES) in a patient, indicating that the correlation between cutaneous features, neurologic symptoms, and respiratory symptoms observed in FES is not causal but rather stochastic (16). This implies that symptomatic sites are directly and causally related only to the site of fat embolism invasion, and that there is no cause-and-effect relationship between individual symptoms. This is at odds with the diagnostic criteria for FES previously proposed by Gurd (5) and Gurd and Wilson (6), who considered the respiratory distress-altered consciousness-bruising rash triad to be the primary criterion for diagnosis. In particular, Gurd argued that patients with FES should be free of brain disease; this raises the question of whether imaging is a negative result. The patient underwent an MRI that showed a large number of scattered foci in the brain, which corresponded to the “star field pattern” described in previous case reports (17, 18). This finding clearly violated Gurd’s diagnostic prerequisites. Consequently, we made a suspected diagnosis of CFES, which was subsequently verified in empirical treatment. In general, there is a dearth of robust clinical evidence and high-quality clinical guidelines for the clinical diagnosis of FES, including thoracic FES and cerebral FES. We propose the utilization of MRI findings of the brain as a clinical basis for the diagnosis of cerebral FES.

Furthermore, we present a case that illustrates the discrepancy between the lesion presentation on MRI images and the patient’s level of consciousness recovery. The initial brain MRI of the patient revealed lesions that exhibited features suggestive of a subacute stage of development (13). The patient’s impaired consciousness demonstrated a complete reversal 7 days after the initial diagnosis. Subsequent brain MRI scans indicated the absorption of the majority of dispersed, multifocal lesions, with a small number of residual lesions persisting. The lesions exhibited a fused and increased volume signal, indicating that the time of arrival of fat emboli to the cerebral vasculature was inconsistent. This suggests that the increased volume lesions may have been a later manifestation of cerebral edema caused by adjacent fat emboli, which is at its peak stage of edema. The enlargement of a few lesions did not impact the improvement in consciousness. It is plausible that the alteration in consciousness was associated with the quantity of fat emboli in the brain and the magnitude of the accumulated lesion volume, rather than the mere enlargement of a few lesions.

While the “star pattern” is highly suggestive of CFES due to its association with disseminated micro-fat emboli, vasogenic edema observed in T2WI may overlap with other cerebral injuries. Thus, the diagnostic value of these MRI features is in combining them with the important clinical manifestations of petechial rash and respiratory distress, and in ruling out other etiologies of traumatic brain injury or metabolic brain that have similar clinical manifestations.

Gurd’s criteria, originally proposed in 1970, remain widely referenced despite lacking formal validation through large-scale prospective studies (5, 6). Their utility lies in their simplicity and ability to consolidate multisystem involvement (respiratory, neurological and cutaneous). In this case, we applied Gurd’s criteria as a pragmatic framework to align with historical diagnostic practices, given the absence of validated alternatives. However, the evolving role of advanced neuroimaging, susceptibility-weighted MRI, may necessitate future revisions to these criteria.

Wang et al. described the case of a 57-year-old male patient who experienced a vehicular accident and subsequently exhibited a gradual return to normal cognitive function 55 days after the diagnosis of cerebral fat embolism (19). However, a significant delay was observed in the detection of nearly complete lesion resorption, as indicated by the brain MRI, which did not manifest until 11 months post-injury (12). This suggests that the resorption of cerebral fat emboli may not always align temporally with the recovery of consciousness in these patients. Our case indicates that the recuperative process of impaired consciousness in young patients with CFES without underlying disease is more abbreviated than the MRI lesion absorption cycle.

### 4.3 Scientific rationale

While our explanations for consciousness recovery are grounded in neuroplasticity and microvascular repair theories, we acknowledge the lack of direct neural recording electroencephalogram (EEG) or functional magnetic resonance imaging (fMRI) data to corroborate these mechanisms. This represents a limitation of our study. Future research incorporating multimodal neuroimaging and electrophysiological assessments could provide deeper insights into the temporal relationship between functional recovery and structural changes.

The principle of this phenomenon can be explained in terms of both neuroplasticity and microvascular repair.

After a cerebral blood vessel suffers an embolic injury from intravascular fat microspheres or fat plugs, the functional areas of the brain innervated by this injured cerebral blood vessel can be functionally compensated by the undamaged functional areas of the brain. This compensatory mechanism is something like a functional reorganisation. In other words, neighbouring or other related brain regions may gradually take over some of the functions for which the damaged region was originally responsible, and consciousness is one of these functions. In the recovery of consciousness, this functional reorganisation may begin to take effect even before a significant lesion improvement can be detected on MRI, allowing the patient’s level of consciousness to be the first to increase while the MRI image still shows the presence of the original lesion.

Another explanation is that after a fat microsphere or fat embolus has embolised a cerebral blood vessel, new blood vessels are created around it to restore blood channels to the area of fat embolism. This establishment of microcirculation may have a positive impact on functional recovery in the early stages, which in turn promotes the restoration of consciousness. However, changes in neoangiogenesis and microcirculation in brain regions may not be immediately recognisable on MRI images because of the limited ability of MRI to detect early subtle changes in the structure and haemodynamics of tiny blood vessels.

### 4.4 Primary “take-away” lessons

Given that MRI lesion uptake is temporally subsequent to the recovery of impaired consciousness, prompt assessment of treatment efficacy should be based on evidence of improvement in clinical manifestations rather than hasty review of the MRI, particularly in patients who have been mechanically ventilated via tracheal intubation.

Moreover, the lesion information from MRI images lags behind the rate of recovery in the level of consciousness. The precise temporal correlation between recovery of the level of consciousness and brain lesion resorption remains unclear, thus necessitating further investigation.

While our follow-up period was limited to 12 days, longer-term MRI and cognitive assessments are warranted to determine whether lesion resolution correlates with sustained recovery or residual deficits. This case underscores the need for extended follow-up in future studies to elucidate the full trajectory of CFES recovery.

### 4.5 Patient perspective

When I started babbling uncontrollably, I was terrified. It was as if I had gone mad. After the ICU doctors gave it some thought, they said I had a cerebral fat embolism syndrome, and it did not take long for my “crazy” state to disappear. After my MRI report came back, I found that the fat embolism in my brain had not been completely absorbed, but I did not want to be discharged immediately. I asked my doctor to let me stay in the hospital until the lesions in my brain were fully absorbed, because I was worried that I might have an accident or that the “madness” symptoms might come back.

## 5 Conclusion

MRI findings should serve as the foundation for diagnosing CFES, rather than being used as a criterion for evaluating discharge. We propose that the combination of the “star pattern” (indicative of microvascular fat emboli) and vasogenic edema on MRI may serve as supportive imaging markers for CFES diagnosis. However, we acknowledge that vasogenic edema alone is non-specific and can occur in other cerebral pathologies, specially traumatic brain injury. Therefore, these findings should be interpreted in conjunction with Gurd’s clinical criteria to enhance diagnostic specificity. The timing of the diagnosis of CFES can be utilized as a validation measure for the diagnosis of CFES, which was conducive to the early and complete recovery from consciousness disorders. Moreover, we found the lesion information from MRI images lags behind the rate of recovery in the level of consciousness. Return of consciousness is a key marker of clinical improvement in CFES. However, therapeutic expectations should also include resolution of systemic manifestations, particularly respiratory dysfunction and cutaneous petechiae, in addition to stabilisation of imaging findings. Discharge decisions should take into account a combination of clinical recovery and trends in lesion regression on magnetic resonance imaging. The patient can then be asked to be followed up after discharge from the hospital, and the end point of the follow-up period can be indicated by observing the complete disappearance of the lesion information shown on MRI.

## Data Availability

The original contributions presented in the study are included in the article/supplementary material, further inquiries can be directed to the corresponding author.
